# The Interdisciplinary Stem Cell Institute’s Use of Food and Drug Administration-Expanded Access Guidelines to Provide Experimental Cell Therapy to Patients With Rare Serious Diseases

**DOI:** 10.3389/fcell.2021.675738

**Published:** 2021-06-08

**Authors:** Aisha Khan, Michael A. Bellio, Ivonne H. Schulman, Allan D. Levi, Bangon Longsomboon, Adriana Brooks, Krystalenia Valasaki, Darcy L. DiFede, Marietsy V. Pujol, Dileep R. Yavagal, Karen E. Bates, Ming-Sing Si, Sunjay Kaushal, Barth A. Green, Kimberly D. Anderson, James D. Guest, Stephen Shelby Burks, Risset Silvera, Andrea J. Santamaria, Anil Lalwani, W. Dalton Dietrich, Joshua M. Hare

**Affiliations:** ^1^Leonard M. Miller School of Medicine, The Interdisciplinary Stem Cell Institute, University of Miami, Miami, FL, United States; ^2^The Miami Project to Cure Paralysis, Leonard M. Miller School of Medicine, University of Miami, Miami, FL, United States; ^3^Katz Family Division of Nephrology and Hypertension, Leonard M. Miller School of Medicine, University of Miami, Miami, FL, United States; ^4^The Department of Neurological Surgery, Leonard M. Miller School of Medicine, University of Miami, Miami, FL, United States; ^5^The Department of Clinical Neurology and Neurosurgery, Leonard M. Miller School of Medicine, University of Miami, Miami, FL, United States; ^6^Section of Pediatric Cardiovascular Surgery, Department of Cardiac Surgery, Michigan Medicine, C.S. Mott Children’s Hospital, Ann Arbor, MI, United States; ^7^Division of Cardiac Surgery, University of Maryland School of Medicine, Baltimore, MD, United States; ^8^Case Western Reserve University School, Cleveland, OH, United States; ^9^Medtronic ST Neurosurgery, Louisville, CO, United States; ^10^Division of Cardiology, Department of Medicine, Leonard M. Miller School of Medicine, University of Miami, Miami, FL, United States

**Keywords:** mesenchymal stem cells, clinical investigation, expanded access IND, schwann cell, single patient IND

## Abstract

The U.S. Food and Drug Administration (FDA) provides guidance for expanded access to experimental therapies, which in turn plays an important role in the Twenty-first Century Cures Act mandate to advance cell-based therapy. In cases of incurable diseases where there is a lack of alternative treatment options, many patients seek access to cell-based therapies for the possibility of treatment responses demonstrated in clinical trials. Here, we describe the use of the FDA’s expanded access to investigational new drug (IND) to address rare and emergency conditions that include stiff-person syndrome, spinal cord injury, traumatic brain stem injury, complex congenital heart disease, ischemic stroke, and peripheral nerve injury. We have administered both allogeneic bone marrow-derived mesenchymal stem cell (MSC) and autologous Schwann cell (SC) therapy to patients upon emergency request using Single Patient Expanded Access (SPEA) INDs approved by the FDA. In this report, we present our experience with 10 completed SPEA protocols.

## Introduction

There are many rare and serious pathological disorders that have no definitive or curative therapeutic modalities. Over the past 20 years, cell-based therapy has been under investigation as an important component of regenerative medicine (Pittenger et al., 2015; Galipeau and Sensebe, 2018; Schulman and Hare, 2018). Autologous and allogenic cell-based therapy, particularly with bone marrow-derived mesenchymal stem cells (BMSCs), holds promise as a therapeutic strategy with a pleotropic mechanism of action, an ability to home in sites of inflammation and injury, and a proven safety record (Bagno et al., 2018; Pittenger et al., 2019; Rieger et al., 2019). In these therapies, primary cells are isolated from donor tissue, expanded, analyzed in a current Good Manufacturing Practice (cGMP) laboratory environment, and delivered to patients. Currently, some cell-based therapeutic products, including MSCs from various tissue sources, are in advanced Phase 3 testing; however, none are approved for clinical use in the United States (Mendicino et al., 2014; Galipeau and Sensebe, 2018). Moreover, most of these potential treatments have not been translated into clinical practice due to the inherent challenges to translational research in rare diseases (Jerusalem et al., 2016). Expanded access has been a practice allowed by the federal law (21CFR312.310), which permits physicians to request experimental treatments for patients on a case-by-case basis, especially in life-threatening situations or in cases where available therapies are lacking (Puthumana et al., 2018). This request is frequently submitted to the U.S. Food and Drug Administration (FDA) as a Single Patient Expanded Access (SPEA) investigational new drug (IND).

The Interdisciplinary Stem Cell Institute (ISCI) at the University of Miami (UM) is a multidisciplinary research facility that supports and develops basic and translational research to advance the utilization of stem cells and the understanding of cellular regenerative mechanisms (Schulman et al., 2018b). Led by skilled and experienced researchers, the Preclinical Program, Clinical Research Cell Manufacturing Program (CRCMP), and Clinical Program adhere to the strictest regulations set forth by the FDA and accredited industry standards (e.g., FACT and AABB). In partnership with the Miami Project to Cure Paralysis (MPCP) at UM, ISCI has strived to develop new strategies for treating the devastating consequences of brain and spinal cord injury (SCI). As such, ISCI has executed several SPEA INDs to accelerate the goal of treating patients in life-threatening situations or in cases without therapeutic options.

To date, ISCI’s SPEA INDs have included the administration of BMSCs and culture expanded Schwann cells (SCs) derived from the sural nerve. MSCs in particular have undergone extensive investigation for several indications such as ischemic and non-ischemic dilated cardiomyopathy (Hare et al., 2012, 2017; Heldman et al., 2014; Florea et al., 2017), idiopathic pulmonary fibrosis (Glassberg et al., 2017), traumatic brain stem injury, and SCI (Santamaria et al., 2018). Furthermore, SCs are excellent candidates for transplantation into patients with peripheral nerve injury (PNI). Since the 1980s, investigators have explored cellular and biologic modalities to enhance peripheral nerve repair (PNR) (Lindsay, 1988; Otto et al., 1987; Rich et al., 1987, 1989). The MPCP has conducted extensive studies demonstrating that cultured SC transplants support PNR in animals with PNI (Guenard et al., 1992; Berrocal et al., 2013). Therefore, transplantation protocols have been developed to investigate the use of SCs in clinical applications (Levi et al., 2016; Gersey et al., 2017).

At ISCI, we have received permission from the FDA for 10 expanded access protocols under seven parent INDs. Herein, we present an overview of our completed SPEA IND studies to provide case-based evidence of safety and feasibility of MSCs and SC cell therapy across various conditions. We have also outlined the regulatory pathway required by the FDA to initiate a SPEA IND for the assessment of novel cell therapies.

## Materials and Methods

### Production of Mesenchymal Stem Cells

The cell manufacturing of the allogeneic and autologous human MSCs used in these INDs was performed at ISCI’s CRCMP, an FDA-registered facility. Briefly, bone marrow was processed using lymphocyte separation medium to prepare the density-enriched mononuclear cells (MNCs). The MNCs were cultured at P0, and MSCs were cultured for three passages. The final passage 3 MSCs were cryopreserved with hespan, 2% human serum albumin, and 5% dimethyl sulfoxide (DMSO) into cryopreservation bags according to the cell dose. All cryopreserved batches of MSCs were tested to ensure that the product passed the established release criterion testing (Table 1). This testing included testing for sterility, which included aerobic, anaerobic, and fungal testing, and found the batches to be free of organisms. The mycoplasma testing, which was performed using PCR, was negative. Cell viability was over 85%, and endotoxin levels were below 0.5 EU/ml. CD105^+^ expression of cells was >85%, and CD45^+^ expression was <2%, measured by flow cytometry (Cytoflex, Beckman Coulter, Brea, CA, United States). Upon request, the appropriate number of released frozen bags was thawed in a 37°C water bath and resuspended in buffer to an appropriate cell concentration in 80 ml of dilution buffer in a sterile bag with Plasma-Lyte A supplemented with 1% human serum albumin. The data collected from each batch of MSCs were documented to pass the release criteria (Table 2). For products to be considered suitable for therapeutic use, surface marker analysis demonstrated positive expression of CD105 >80% and CD45 <2% prior to cryopreservation; no growth of anaerobic, aerobic, and fungal contamination was reported; endotoxin levels were ≤ 5 EU/patient kg; cell viability was measured to be ≥70%; mycoplasma RNA was undetected by PCR; and no organisms were seen by Gram stain.

**TABLE 1:** Release specifications of cryopreserved and thawed MSC products.

**Assay**	**Sample**	**Acceptance criteria**
**Release specifications of cryopreserved products**		
Sterility	Cryopreserved product	Final result: Negative or no growth
Endotoxin	Cryopreserved product	≤5EU/patient kg
Viability	Cryopreserved product	≥70%
CD105/CD45 Cell Quantitation	Cryopreserved product	CD105^+^ >80%/ CD45^+^ >2%
Mycoplasma PCR	Cells in Conditioned Media prior to cryopreservation	Negative by PCR
**Release specifications of thawed products**		
Sterility	Thawed product	No growth
Sterility: Gram stain	Thawed product	No organisms seen
Endotoxin	Thawed product	≤5EU/patient kg
Cell count	Thawed product	Dose specific
Viability	Thawed product	≥70%

**TABLE 2:** Release criteria analysis of final MSC products.

**IND number**	**Pre cryo flow**	**Pre-cryo sterility**	**Pre-cryo mycoplasma**	**Pre-cryo viability**	**Post-thaw sterility**	**Post-thaw endotoxin**	**Post-thaw viability**
16093	Not completed	No growth	Negative	100%	No growth	≤0.5 EU/mL	83%
15969	Not completed	No growth	Negative	95%	No growth	≤0.5 EU/mL	78% 84% 83%
15699	Not completed	No growth	Negative	78%	No growth	≤0.5 EU/mL	88%
16045	CD105^+^ 99.87% CD45^+^ 0.42%	No growth	Negative	94%	No growth	≤0.5 EU/mL	89%
17911	CD105^+^ 99.72% CD45^+^ 0.80%	No growth	Negative	94%	No growth	≤0.5 EU/mL	94%
18102	CD105^+^ 99.75% CD45^+^ 0.93%	No growth	Negative	97%	No growth	≤0.5 EU/mL	93%
14856	CD105^+^ 99.75% CD45^+^ 0.93%	No growth	Negative	97%	No growth	≤0.5 EU/mL	88%
14856	CD105^+^ 99.75% CD45^+^ 0.93%	No growth	Negative	97%	No growth	≤0.5 EU/mL	90%

### Production of Stem Cells

Autologous SCs were derived from the sural and sciatic nerves as previously described (Levi et al., 2016). In brief, SCs were isolated from a nerve biopsy approximately 5–10 cm in length and passaged up to three times depending on the number of cells needed for infusion and cryopreserved at harvest for LN2 storage. The sural and sciatic nerves were processed similarly; however, sciatic nerves were delivered in small segments, as they were derived from nerve tips rescued from the injured tissue. Several controls are employed throughout the manufacturing progression to ensure that the product is essentially free of process-related contaminants, including mitogens, laminins, and bovine residuals. For products to be considered suitable for therapeutic use, cell viability was measured to be >70%; purity was determined to be ≥90% CD271^+^ cells by flow cytometry analysis; no growth of anaerobic, aerobic, and fungal contamination was reported; mycoplasma RNA was undetected by PCR; and no organisms were seen by Gram stain. Two Gram stains are performed using the culture medium and final wash during preparation for transplant. After all necessary results are available, the SCs are cleared for transplantation (Tables 3, 4).

**TABLE 3:** Release specifications of cryopreserved and thawed SC products.

**Assay**	**Sample**	**Acceptance criteria**
**Release specifications of fresh products**		
Viability	Fresh product	≥70%
Purity	Fresh product	≥90% CD271^+^
Mycoplasma PCR	Cells in conditioned media prior to cryopreservation	Negative
Sterility	Fresh product	No growth
Sterility: gram stain	Thawed product	No organisms seen

**TABLE 4:** Release criteria analysis of final SC products.

**IND#**	**Collected on the day of Tx**				**Collected 3 days prior to Tx**		**Collected post-transplant**
	**Cell count**	**Viability**	**Gram stain**	**Purity**	**MycoplasmaPCR**	**Sterility**	**Mycoplasma–culture**
14856	271 × 10^6^	97.8%	Negative	98.4% CD271^+^	Negative	No growth	Negative
14856	10.0 × 10^6^ (Sciatic)	99.3% (sciatic)	Negative	96.5% CD271^+^ (sciatic)	Negative	No growth	Negative
	18.8 × 10^6^ (sural)	99.8% (sural)		90.9% CD271 + (sural)			

## Results

### Protocol #1—Single Patient Expanded Access Investigational New Drug to Deliver Allogeneic Human Mesenchymal Stem Cells to a Woman With Stiff-Person Syndrome

Stiff-person syndrome (SPS) is a rare neurological disorder in which patients exhibit rigidity, stiffness, stiff legs, waddling gait, hyperreflexia, and spasms (Gershanik, 2009). At disease onset, symptoms may be infrequent and mild in character but then progress to decreased activities of daily living and followed by severe stiffness to the point where patients are unable to move their head from side to side (Gershanik, 2009). The pathophysiology of this autoimmune disease results from autoantibodies, and dysfunction of GABAergic neurons has been linked as a causative mediator of SPS (Chang et al., 2013). Current treatment options for SPS are highly limited with variable clinical efficacy of immune globulin therapy (Dalakas et al., 2001). In many cases, patients are treated with benzodiazepines (Pagano et al., 2014) and intravenous (IV) diazepam administration (Bhatti and Gazali, 2015). Despite treatment with the available therapeutics, disability and the severe suppression of the quality of life are common (Baizabal-Carvallo and Jankovic, 2015). Furthermore, in severe cases, spontaneous neurological events can lead to high rates of mortality (Barker et al., 1998; Duddy and Baker, 2009). Using a SPEA IND, we delivered allogeneic MSCs to a 71-year-old woman who had a history of anti-GAD-mediated SPS with chronic symptoms of vitiligo, anxiety, tension and involuntary muscle contractions, and severe focal pain. Previous treatment rounds of IV immunoglobulin and botulinum toxin injections provided minimal improvements. Therefore, we completed IV administration of 200 × 10^6^ MSCs (Table 5). No adverse side effects were noted after the infusion; however, the patient failed to complete the requested follow-ups and was therefore withdrawn from the study.

**TABLE 5:** Outline of single patient INDs investigating mesenchymal stem cells.

**Protocol #1**	**FDA IND # 16093**
Sponsor	Interdisciplinary Stem Cell Institute, University of Miami Miller School of Medicine ISCI
Investigator	Joshua Hare, MD
Study institute	Interdisciplinary Stem Cell Institute, University of Miami Miller School of Medicine University of Miami
Name of therapy	Bone marrow derived human Mesenchymal stem cells (MSCs)
Study title	Single patient expanded access IND to deliver allogeneic mesenchymal stem cells (Allo-hMSCs) to a woman with Stiff Person Syndrome
Design and investigational plan	Allo-hMSCs source manufactured by the CRCMP. One dose of intravenous infusion (IV) of 200 × 10^6^ MSCs.
Patient population	Single patient
Route of administration	IV
Study follow-up	Patient withdrawn after treatment
**Protocol #2**	**FDA IND # 15969**
Sponsor	Interdisciplinary Stem Cell Institute, University of Miami Miller School of Medicine
Investigator	James Guest, MD, Ph.D.
Study institute	Miami Project to Cure Paralysis
Name of therapy	Bone marrow derived human mesenchymal stem cells (MSCs)
Study title	Single patient expanded access IND to deliver allogenic and autologous bone marrow mesenchymal stem cells (BMSCs) to the spinal cord of a young woman with a subacute complete C3 cervical spinal cord injury (SCI).
Design and investigational plan	Allo-hMSCs and auto-MSCs source manufactured by the CRCMP. Dose # 1–Intrathecal (IT) administration of 1 × 10^6^ Allo-MSCs/kg body weight. Dose # 2–IT administration of 1 × 10^6^ Auto-MSCs/kg body weight. Dose # 3–IT administration of 1 × 10^6^ Auto-MSCs/kg body weight.
Patient population	Single patient
Route of administration	IT
Study follow-up	26 months
**Protocol #3**	**FDA IND # 15699**
Sponsor	Interdisciplinary Stem Cell Institute, University of Miami Miller School of Medicine
Investigator	Dileep R. Yavagal, MD
Study institute	Interventional Neurology, University of Miami Hospital
Name of therapy	Bone marrow derived human mesenchymal stem cells (MSCs)
Study title	A physician IND protocol for the compassionate use of allogeneic peripheral IV to a subject with traumatic brain stem injury.
Design and investigational plan	Allo-hMSCs source manufactured by the CRCMP. Intravenous infusion (IV) administration of 100 × 10^6^ MSCs.
Patient population	Single patient
Route of administration	IV
Study follow-up	30 days
**Protocol #4**	**FDA IND # 16045**
Sponsor	Interdisciplinary Stem Cell Institute, University of Miami Miller School of Medicine
Investigator	Ming-Sag Si, MD
Study institute	University of Michigan Health System
Name of therapy	Bone marrow derived human mesenchymal stem cells (MSCs)
Study title	Single patient expanded access IND to deliver allogeneic mesenchymal stem cells (Allo-hMSCs) to an 8 month old with End-stage Heart Failure (heterotaxy syndrome).
Design and investigational plan	Allo-hMSCs source manufactured by the CRCMP. Intramyocardial injections of 2.5 × 10^5^ cells per kg of recipient (1.95 × 10^6^). The eight open intramyocardial injections during a sternotomy at 100 mL volume per injection using a 27-gauge needle syringe.
Patient population	Single patient
Route of administration	Intramyocardially
Study follow-up	1 months
**Protocol #5**	**FDA IND # 17911**
Sponsor	Interdisciplinary Stem Cell Institute, University of Miami Miller School of Medicine
Investigator	Eric Peterson, MD
Study institute	Department of Neurology, University of Miami Miller School of Medicine
Name of therapy	Bone Marrow Derived Human Mesenchymal Stem Cells (MSCs)
Study title	V1:Single patient expanded access IND to deliver allogeneic mesenchymal stem cells (allo-MSCs) to a subject experienced a devastating brain hemorrhage. V2: Amendment for expanded access for ischemic stroke: Physician IND protocol for the compassionate use of allogeneic MSC infusion to a subject with a basilar artery occlusion resulting in locked-in syndrome. V3: Amendment for expanded access for ischemic stroke: Physician IND protocol for the compassionate use of allogeneic MSC infusion to a subject with a large left middle cerebral artery stroke.
Design and investigational plan	Allo-hMSCs manufactured by the CRCMP. Intravenous (IV) administration of 200 × 10^6^ MSCs.
Patient population	Single patient
Route of administration	IV
Study follow-up	6–12 months
**Protocol #6**	**FDA IND # 18102**
Sponsor	Interdisciplinary Stem Cell Institute, University of Miami Miller School of Medicine
Investigator	Dileep R. Yavagal, MD
Study institute	Interventional Neurology, University of Miami Hospital
Name of therapy	Bone Marrow Derived Human Mesenchymal Stem Cells (MSCs)
Study title	Single patient expanded access IND for intravenous delivery of MSC to a patient with high-grade aneurysumal subarachnoid hemorrhage.
Design and investigational plan	Allo-hMSCs manufactured by the CRCMP. Intravenous (IV) administration of 100 × 10^6^ MSCs.
Patient population	Single patient
Route of administration	IV
Study follow-up	12 months

### Protocol #2: Single Patient Expanded Access Investigational New Drug to Deliver Allogeneic and Autologous Bone Marrow-Derived Mesenchymal Stem Cells to the Spinal Cord of a Young Woman With a Subacute Complete C3 Cervical Spinal Cord Injury

There are currently no curative treatments for SCI, and the prognosis for mortality after upper level cervical SCI is higher than SCI at the lower levels. Patients with chronic complete C3 tetraplegia have limited potential for motor and sensory recovery below the injury level and require life-long assistance with activities of daily living. The subject of this IND was a 24-year-old athlete who suffered from a bilateral C3/4 spinal dislocation with a motor–sensory complete C3 American Spinal Injury Association (ASIA) SCI with no arm and leg function (Santamaria et al., 2018). Over a 1-year period, we delivered three doses of MSCs by intrathecal (IT) administration at 1 × 10^6^ MSCs/kg body weight (50 × 10^6^ cells for this 50-kg patient) at 114, 183, and 307 days post injury (Table 5). No serious adverse effects were reported.

Twenty-six months after the first infusion, the subject had considerable recovery of surface touch and pressure sensation. Detailed neurophysiological testing detected connections in the scapular muscles, deltoid, rectus abdominis, and biceps, most of which improved at each assessment. Unusual plasticity revealed the formation or unmasking of connections between the breathing centers and arm muscles such that each deep breath triggered electromyography (EMG) activity. Given the paucity of electrophysiological studies in high-level tetraplegics, it is unknown if such plasticity occurs naturally or was related to BMSC infusion.

### Protocol #3: A Physician Investigational New Drug Protocol for the Compassionate Use of Allogeneic Mesenchymal Stem Cells via Peripheral Intravenous to a Subject With Traumatic Brain Stem Injury

The subject of this IND was a 58-year-old male with a history of hypertension who had fallen and was found to have a traumatic brain stem injury. Initial neurological evaluation by CT of the brain revealed bilateral subdural hematomas, 10 mm in size, as well as subdural blood along the falx; and the patient was taken to the emergency room for a bilateral craniotomy. Furthermore, a postoperative CT of the brain showed a Duret hemorrhage of the brainstem. He received a single IV administration of 100 × 10^6^ allogeneic MSCs 4 days post-brain injury (Table 5). At 7 days post-MSC treatment, the patient remained a 4 on the Glascow Coma Scale with fixed pupils. Follow-up imaging revealed extensive bi-hemispheric injury as well as severe injury to the midbrain and upper pons. Prior to 30 days post-MSC treatment, the family chose to withdraw care, and the patient was pronounced deceased.

### Protocol #4: Single Patient Expanded Access Investigational New Drug to Deliver Allogeneic Mesenchymal Stem Cells to an 8-Month-Old With Complex Congenital Heart Disease

Congenital heart disease and complications from the surgical procedures to correct them can lead to end-stage heart failure in neonates and infants. The outcome of these patients is poor, and heart transplant is the standard therapeutic option. However, the waitlist time for transplantation may be too long for these young, high-risk patients; and other treatments of the congested state of the heart must be considered. The subject of our IND was an 8-month-old with heterotaxy syndrome, levocardia, double outlet right ventricle, ventricular septal defect, sub-valvar and valvar pulmonary stenosis, right aortic arch with mirror image branching, and patent ductus arteriosus. This patient underwent systemic to pulmonary shunt in the newborn period followed by a complex biventricular repair consisting of a Senning–Rastelli double switch procedure. The patient was unable to wean from cardiopulmonary bypass and was transitioned to venoarterial extracorporeal membrane oxygenation (ECMO) support. Her repair was revised to relieve a coronary artery distortion from a nearby suture line of the right ventricle to pulmonary artery conduit. Despite reestablishment of coronary flow, her morphologic left ventricular function remained depressed, and she was converted to an extracorporeal left ventricular assist device (LVAD) as a bridge to heart transplantation 2 weeks later. Given the poor prognosis, extended waitlist time, and significant complication rate of mechanical circulatory support in these patients, we considered the use of MSC therapy to improve ventricular function enough to wean from the LVAD. We have demonstrated clinical benefits of MSC therapy in adults with heart failure and data suggestive of a positive benefit in promoting cardiac regeneration and improvement in function (Heldman et al., 2014). Therefore, given the potential for mortality, we proposed to deliver intramyocardial doses of allogeneic MSCs at 2.5 × 10^5^ cells per kg (1.99 × 10^6^ total cells to this 7.5-kg patient, Table 5). The cells used for this study were shipped overnight to the University of Michigan where they were injected into the free wall of the left ventricle under the supervision of ISCI staff at the time of delayed sternal closure from her LVAD implantation. The patient tolerated this procedure well. Postoperatively, she continued on LVAD support and required multiple LVAD circuit changes for thrombus formation, while the left ventricle function remained depressed. Approximately 1 month later, she developed sepsis and significant abdominal aortic thrombus formation and subsequently expired.

### Protocol #5v1: A Physician Investigational New Drug Protocol for the Compassionate Use of Allogeneic Mesenchymal Stem Cell Infusion to a Subject With a Large Right Middle Cerebral Artery Stroke

The subject of this IND wa m a gunshot wound to the psa 69-year-old Hispanic female without significant past medical history who presented with visual disturbances and was diagnosed with a giant right internal carotid artery (ICA) aneurysm measuring 2.5 × 3 cm compressing her optic chiasm. Following the pipeline embolization procedure for the right ICA aneurysm, the subject developed a significant right middle cerebral artery (MCA) ischemic stroke that caused her left hemiparesis and eyelid apraxia. Family consent was obtained, and the subject received IV 100 × 10^6^ allogeneic MSCs 5 days post-stroke (Table 5). The subject was followed weekly until hospital discharge and at 30, 90, and 180 days thereafter. Despite initial neurological worsening resulting in decompressive hemicraniectomy, this subject showed cognitive and sensory improvements over a 6-month period. At 180 days post-MSC treatment, modified Rankin Score (mRS) and National Institutes of Health Stroke Scale (NIHSS) assessments demonstrated unchanged left hemiplegia, but the subject was able to answer questions correctly, was able to eat, and showed improvement in sensory deficits. Subsequent to the 180-day follow-up, the subject suffered an intraparenchymal hemorrhage that ultimately led to her death 8 months following the initial aneurysm and MSC treatment. Serious adverse events related to the investigational product were not observed.

### Protocol #5v2: Amendment for Expanded Access for Ischemic Stroke: Physician Investigational New Drug Protocol for the Compassionate Use of Allogeneic Mesenchymal Stem Cell Infusion to a Subject With a Basilar Artery Occlusion Resulting in Locked-in Syndrome

Due to similarities in severity of stroke ischemia and lack of therapeutic options, the existing protocol for IND #5 was amended to include two more subjects. The subject of this amendment is a 76-year-old Haitian male with past medical history of hypertension, dyslipidemia, type 2 diabetes, and heart failure with reduced ejection fraction who presented with Basilar stroke and underwent mechanical thrombectomy with stent placement. Immediately postoperatively, the patient showed movement in his face and extremities but subsequently suffered postoperative stent occlusion and became clinically locked-in. Locked-in syndrome is a rare neurological disease in which the subject is wakeful and aware but quadriplegic. Given that there is no cure for locked-in syndrome, expanded access use of IV MSCs is an experimental option. For this subject, 200 × 10^6^ allogeneic MSCs were administered by IV 5 days post-stroke (Table 5). This subject remains stable in a supported care environment but has not shown any improved neurological function over a 6-month period. Adverse events during long-term follow-up included upper limb edema and open wound infections, neither of which is related to the investigational product.

### Protocol #5v3: Amendment for Expanded Access for Ischemic Stroke: Physician Investigational New Drug Protocol for the Compassionate Use of Allogeneic Mesenchymal Stem Cell Infusion to a Subject With a Large Left Middle Cerebral Artery Stroke

The subject of the second and final amendment to the existing protocol #5 is a 31-year-old Caucasian male with past medical history of benign ameloblastoma of the left posterior mandible and a former smoker. Following extubation after a tumor resection and bony transplantation procedure, the patient exhibited left MCA syndrome. CT/CT perfusion (CTP) imaging showed a left M1 occlusion with large core infarct and was determined not to be a candidate for mechanical thrombectomy. Upon examination following decompressive hemicraniectomy, the patient had left hemiplegia and expressive aphasia. Expanded access of IV MSCs was considered given that medical management was the only therapeutic option and that the patient is a young, left-handed male who could achieve meaningful recovery. The patient received 200 × 10^6^ MSCs IV 5 days post-stroke (Table 5). This patient demonstrated partial neurological recovery and is still being followed up. He has regained partial strength in both upper and lower affected limbs, is able to walk independently with a cane, and demonstrates improved speech and communication. Family reported adverse events include three seizures over a 6-month period. The first seizure was observed in the hospital following bone flap replacement surgery approximately 30 days post-MSC treatment. Two more seizures were observed more than 90 days post-MSC treatment while traveling abroad and shortly thereafter. It was also reported that this subject received intra-nasal autologous adipose-derived stem cell therapy while traveling abroad in the months prior to our 180-day follow-up visit.

### Protocol #6: Single Patient Expanded Access Investigational New Drug for Intravenous Delivery of Mesenchymal Stem Cell to a Patient With High-Grade Aneurysmal Subarachnoid Hemorrhage

The subject of this IND was an 80-year-old man who experienced a sudden onset of severe headache, nausea, and unconsciousness due to an aneurysmal subarachnoid hemorrhage (SAH) (Brunet et al., 2019). A CT scan demonstrated diffuse SAH and hydrocephalus. According to the Hunt and Hess scale, he had grade 5 SAH. An angiogram revealed a large irregular broad-based aneurysm of the basilar tip. The aneurysm was repaired with a stent-assisted coiling technique, but his neurological condition remained the same. Despite the decrease in case-fatality rate after SAH from improved detection technologies, the pathophysiologic processes of SAH lead to dismal functional outcomes. Therefore, an emergency compassionate use was authorized to deliver an IV dose of 100 million MSCs (Table 5). At the conclusion of the study period, 12 months following MSC infusion, the patient achieved rapid and favorable recovery as documented by an increased Rankin scale score of 3 following the high-grade SAH.

### Protocol #7: Transplantation of Autologous Human Schwann Cells for Peripheral Nerve Repair—Compassionate Use

The subject of this single patient IND was a 25-year-old female who suffered from a traumatic sciatic nerve injury with a significant gap due to a spinning boat propeller hitting her right posterior thigh. At the time of injury, she was immediately rushed to the trauma operating room to control the bleeding from life-threatening hemorrhage from her lower extremity. She later underwent a total of four additional surgeries including thigh wound washout and debridement of necrotic muscle, Achilles tendon repair, anterior quadriceps washout and tendon repair, and skin grafting to her posterior thigh. The application of autologous SCs in combination with nerve autografts was hypothesized to offer a potential novel therapeutic option. Autologous SCs were derived from the traumatized sciatic nerve stumps harvested at the time of injury as well as a sural nerve biopsy taken at the time of Achilles tendon repair. Surgery to repair the sciatic nerve and transplant the autologous SCs occurred 30 days post injury. During the surgery, multiple portions of a nerve graft were placed to span the sciatic nerve stumps injury and sutured with 7-0 prolene. This repair was supplemented with 29 × 10^6^ autologous SCs surrounded by an FDA-approved collagen matrix, DuraGen (Table 6). At the time of injury, the patient had lost all sensory and motor function of the sciatic nerve distal to the innervation of the knee flexors. However, 15 months post-surgery, the patient’s neurological exam indicated sensitivity to pin prick below the knee. Definitive motor evidence of nerve regeneration was noted at 18 months by robust ankle plantarflexion [grade 4/5 using Medical Research Council (MRC) scale] with some dorsiflexion at 24 months. At 5 years post-transplant, her neurological recovery has been maintained with no evidence of tumor or development of a pain syndrome.

**TABLE 6:** Outline of single patient INDs investigating schwann cells.

**Protocol #7**	**FDA IND # 14856**
Sponsor	Miami Project to Cure Paralysis, University of Miami Miller School of Medicine
Investigator	Allan Levi, MD, Ph.D
Study institute	Miami Project to Cure Paralysis
Name of therapy	Autologous sural nerve and sciatic nerve derived schwann cells
Study title	Transplantation of autologous human schwann CELLS (ahSCs) for peripheral nerve repair (PNR)—compassionate use.
Design and investigational plan	The cells for the autologous sural nerve and sciatic nerve derived schwann cells manufactured by CRCMP. A single dose of 19 × 10^6^ sural nerve derived ahSCs and a single dose of 10 × 10^6^ sciatic nerve derived ahSCs.
Patient population	Single subject with acute sciatic nerve transection with significant loss of peripheral nerve tissue at posterior mid-thigh level.
Route of administration	Injury site
Study follow-up	5 years
**Protocol #8**	**FDA IND # 14856**
Sponsor	Miami Project to Cure Paralysis, University of Miami Miller School of Medicine
Investigator	Allan Levi, MD, PhD
Study institute	Miami project to cure paralysis
Name of therapy	Autologous sural nerve derived schwann cells
Study title	Emergency expanded access request to IND to use autologous human schwann cells (ahSCs) augmentation of nerve autograft repair in a single patient with severe peripheral nerve injury.
Design and investigational plan	The cells for the autologous sural nerve derived schwann cells manufactured by CRCMP. Single dose of 110 × 10^6^ sural nerve derived ahSCs.
Patient population	Single subject with acute sciatic nerve injury at mid-thigh level
Route of administration	Injury site
Study follow-up	3 years

### Protocol #8: Amendment for Expanded Access for Peripheral Nerve Injury

The subject of this IND was a 30-year-old female who suffered from a gunshot wound to the posterior right midthigh. In this injury, bullet fragments became lodged within the sciatic nerve resulting in the loss of sensation in the sciatic distribution of the right lower extremity. Immediately post injury, there was no surgical debridement of the wound; however, a sural nerve biopsy was collected for SC manufacturing. Cell transplantation of 100 × 10^6^ SCs within Duragen was performed 41 days after the injury in conjunction with three 5-cm nerve grafts (Table 6). There were no reported postoperative complications with the sciatic nerve repair or SC transplantation. Over the course of the 12-month follow-up, the patient experienced significant improvements in toe dorsiflexion, foot plantar flexion, and inversion. Furthermore, her neuropathic pain gradually improved throughout the course of follow-up.

At 3 years after injury, the neurological exam demonstrated normal sensation to light touch and pin prick in the distribution of the superficial peroneal nerve branch. Impaired sensation was present in the sural, deep fibular, and medical calcaneal branches. At this time, we observed significant improvement in toe dorsiflexion (4/5), foot dorsiflexion (4/5), and plantar flexion (4/5), eversion (5/5), and inversion (4/5). MRI at 3 years post injury demonstrated continuity of the graft and no evidence of tumor or neuroma formation.

## Discussion

Cell therapy has undergone extensive experimental investigation for several serious medical conditions. In the SPEA INDs reported here, MSCs and SCs were also applied to a range of life-threatening and traumatic injuries. The exploration of clinical safety and effectiveness of new drugs and therapies is often limited by strict clinical trial guidelines, costs, and access to subjects. Therefore, expanded access, also called “compassionate use,” provides a pathway for patients to gain access to investigational drugs, biologics, and medical devices used to diagnose, monitor, or treat patients with serious diseases or conditions for which there are no comparable or satisfactory therapy options available outside of clinical trials. The FDA facilitates approval of the expanded access process; however, access to investigational treatments requires not only the FDA’s review and authorization but also the active involvement and cooperation of other parties, including drug companies and healthcare providers, in order to be successful.

The goal of these studies was to utilize safe cell therapies in situations where treatment options are limited. Furthermore, this compiled analysis demonstrates the feasibility of completing single patient studies while adhering to personalized and standard treatment associated with traumatic and life-threatening disorders. Although results are varied, our experience has shown that SPEA INDs are a viable experimental therapy for most of these conditions. Here, we share our experiences for physicians to have an understanding of the mechanism available for them to try a therapy that not only is approved but also has safety profile with the FDA in the form of phase I, II, or III INDs.

### Food and Drug Administration Guidelines and Requirements of Single Patient Expanded Access Investigational New Drug

The FDA facilitates the approval of the expanded access process for both emergency and non-emergency manner cases (Knoepfler, 2015; Jarow et al., 2016, 2017; Folkers and Bateman-House, 2018). In both scenarios, successful access to new drug investigational treatments must first be subjected to the FDA’s review and authorization through an IND application. There also must be active involvement and cooperation of other parties under FDA regulations 21 CFR 312.305 and 21 CFR 312.310. After the approval of a parent IND application, these regulations permit an investigational drug to be used for emergency treatment of an individual patient by a licensed physician, under the following circumstances: (1) the patients have a serious or immediate life-threatening disease or condition, which means a stage of disease in which there is a reasonable likelihood that death will occur within a matter of months or in which premature death is likely without early treatment. Serious disease or condition also means a disease or condition associate with morbidity that has substantial impact on day-to-day functioning. (2) There is no comparable or satisfactory alternative therapy to diagnose, monitor, or treat the disease or condition. (3) The probable risk to the patient from the investigational drug is not greater than the probable risk from the disease or condition. (4) The expanded use of the investigational drug for the requested treatment will not interfere with the initiation, conduct, or completion of clinical investigations that could support marketing approval of the product.

For all non-emergency cases, the FDA and Institutional Review Board (IRB) must receive a written application (Form FDA 3926) in advance (Folkers and Bateman-House, 2018). However, when a patient’s condition is dire and there is insufficient time to submit a written application, a physician can request an emergency expanded access from the FDA over the phone. At that time, the FDA will decide whether the expanded access request can proceed. The IRB may also review an application as an emergency; follow-up written documentation must be submitted within the time-period specified in the FDA guidelines (within 5 days of receiving therapy). Following IRB approval for non-emergency and acknowledgment for emergency, the expanded access application and a letter of authorization are submitted from the drug manufacturer to the FDA within 15 business days. In a non-emergency case, the processes are not different in substance, only in timing. All of our completed INDs are examples of emergency expanded access INDS, as the patients conditions were deemed dire based on severity and lack of treatment options.

### Review of Mesenchymal Stem Cell Safety in Our Investigational New Drugs

Allogeneic MSC products are prime candidates for use in SPEA INDs because autologous cell therapy required a lengthy production time from tissue harvest and cell expansion to final product infusion. Utilizing a banked stock of allogeneic MSCs is a much more feasible option when timing is a critical limitation. Importantly, we have shown our MSC allogeneic bank to be not only safe but also potentially more effective than autologous products in reversing remodeling and improving cardiac function in patients with dilated cardiomyopathy (Premer et al., 2015; Fontaine et al., 2016; Hare et al., 2017).

The results of our SPEA IND experience meet our primary objective to demonstrate the safety of allogeneic MSC treatment in all the tested emergency situations. However, due to the various disease severities, it is difficult to perform secondary analysis of product efficacy within individual cases. In our patient cases with SCI and ischemic stroke (protocol 2, protocol 5v1, and protocol 5v3), improved outcomes were documented after treatment. However, patients in many of our studies were withdrawn, thus preventing follow-ups and efficacy evaluations.

### Review of Schwann Cell Safety in Our Investigational New Drugs

The completed SPEA INDs investigating the transplantation of autologous SCs for PNR meet our primary objective for the demonstration of safety. Furthermore, collected results of these trial further highlight the potential for efficacy in the recipient patients. In both trials, neurological examination of the left foot showed significant improvement in such motor actions like flexion, eversion, and extension at all the joints except for foot inversion. However, sensory modality only showed effectiveness in deep fibular nerve.

### Advantages and Limitations of Single Patient Expanded Access Investigational New Drug Studies

There are several advantages for using a SPEA IND pathway for the development of clinical treatments. The primary objective of SPEA INDs is to demonstrate safety and feasibility of the treatment protocol in the at-risk patient population. However, secondary outcomes regarding patient outcome may be useful for the development of large-scale clinical trials to reach a broader population. For instance, clinical trials are not only costly but also difficult to conduct (Hare et al., 2013; Schulman et al., 2018a). Moreover, the limited window for treatment time in some rare and life-threatening conditions can result in clinical development delays (Jin et al., 2017). The insights acquired from SPEA INDs may be informative to guide therapeutics development leading to eventual implementation to clinical practice (Hogan, 2016). Furthermore, these studies provide access that supersedes traditional drug development. As individualized studies, SPEA INDs offer a viable opportunity for medical practitioners to advance personalized clinical practice. We are not implying that SPEA has the value of well-designed clinical trials with control groups but that the process may accelerate therapeutics development in well-selected situations. Data collected from SPEA of rare or life-threatening disease could be particularly useful in the clinical development therapies under the Regenerative Medicine Advanced Therapy (RMAT) pathway, where preclinical models are limited. However, the extensive public attention to the SPEA IND has raised some concerns within the scientific research field that these IND studies could potentially interfere with the progressive placebo-controlled clinical trials. The most common challenge owes its basis to the fact that these studies could result in a decrease in the number of subjects available for recruitment into clinical trial studies, which could be a significant issue, especially for the specific pathological conditions of low prevalence such as SPS.

It takes an average of 10 years to achieve market approval for biologics and drugs, and over 90% of Phase I clinical trials fail to obtain approval (Hare et al., 2013; Schulman et al., 2018a). For patients with life-threatening or severely debilitating disease and limited treatment options, the right to access investigational drugs prior to approval has become a priority. Regulatory and legislative efforts have resulted in earlier access to investigational drugs, through the FDA Expanded Access programs. Our collective experience documents the feasibility and safety of using MSCs and SCs on rare and life-threatening conditions.

## Data Availability Statement

The original contributions presented in the study are included in the article/supplementary material, further inquiries can be directed to the corresponding author/s.

## Ethics Statement

The studies involving human participants were reviewed and approved by the University of Miami IRB and Jackson Memorial Hospital IRB. Written informed consent to participate in this study was provided by the participants’ legal guardian/next of kin.

## Author Contributions

AK, MB, IS, AL, BL, AB, KV, DDif, MP, DY, KB, MS-S, SK, BG, KA, JG, SB, RS, AS, AL, WD, and JH contributed to experimental design, completing single patient cases, data collection, data interpretation, data reporting, and writing. All authors contributed to the article and approved the submitted version.

## Disclaimer

IS has no disclosures and contributed to this manuscript in her personal capacity while at the University of Miami. IS now is employed by the National Institutes of Health (NIH). The opinions expressed in this article are the author’s own and do not reflect the view of the NIH, the Department of Health and Human Services, or the United States government.

## Conflict of Interest

AK discloses a relationship with AssureImmune Cord Blood Bank and Aceso Therapeutic that includes equity. JH reports having a patent for cardiac cell-based therapy and holds equity in Vestion Inc. and maintains a professional relationship with Vestion Inc. as a consultant and member of the Board of Directors and Scientific Advisory Board. JH was the Chief Scientific Officer, a compensated consultant and advisory board member for Longeveron and holds equity in Longeveron. JH was also the co-inventor of intellectual property licensed to Longeveron. AL, WD, JG were disclosed a relationship with Aceso Therapeutic that includes equity. DY was a consultant for Cerenovous, Deck Therapeutics, GLG Consulting, Guidepoint Consulting, Inneuroco, Inc., Mosaic Consulting, and Poseydon Medical, LLC. He is both a consultant and a steering committee member for Medtronic, Rapid Medical, and Vascular Dynamics. He is also on the Medical Advisory Board for Neuralanalytics. The remaining authors declare that the research was conducted in the absence of any commercial or financial relationships that could be construed as a potential conflict of interest.

## References

[B1] Bagno L. Hatzistergos K. E. Balkan W. Hare J. M. (2018). Mesenchymal stem cell-based therapy for cardiovascular disease: progress and challenges. *Mol. Ther.* 26 1610–1623. 29807782 10.1016/j.ymthe.2018.05.009 PMC6037203

[B2] Baizabal-Carvallo J. F. Jankovic J. (2015). Stiff-person syndrome: insights into a complex autoimmune disorder. *J. Neurol. Neurosurg. Psychiatry* 86 840–848. 10.1136/jnnp-2014-309201 25511790

[B3] Barker R. A. Revesz T. Thom M. Marsden C. D. Brown P. (1998). Review of 23 patients affected by the stiff man syndrome: clinical subdivision into stiff trunk (man) syndrome, stiff limb syndrome, and progressive encephalomyelitis with rigidity. *J. Neurol. Neurosurg. Psychiatry* 65 633–640. 10.1136/jnnp.65.5.633 9810930 PMC2170335

[B4] Berrocal Y. A. Almeida V. W. Gupta R. Levi A. D. (2013). Transplantation of Schwann cells in a collagen tube for the repair of large, segmental peripheral nerve defects in rats. *J. Neurosurg.* 119 720–732. 10.3171/2013.4.jns121189 23746104

[B5] Bhatti A. B. Gazali Z. A. (2015). Recent advances and review on treatment of stiff person syndrome in adults and pediatric patients. *Cureus* 7:e427. 26848416 10.7759/cureus.427 PMC4727915

[B6] Brunet M. C. Chen S. H. Khandelwal P. Hare J. Starke R. M. Peterson E. C. (2019). Intravenous stem cell therapy for high grade aneurysmal subarachnoid haemorrhage: case report and literature review. *World Neurosurg.* 128 573–575. 10.1016/j.wneu.2019.04.055 30981798

[B7] Chang T. Alexopoulos H. McMenamin M. Carvajal-Gonzalez A. Alexander S. K. Deacon R. (2013). Neuronal surface and glutamic acid decarboxylase autoantibodies in nonparaneoplastic stiff person syndrome. *JAMA Neurol.* 70 1140–1149. 10.1001/jamaneurol.2013.3499 23877118 PMC6055982

[B8] Dalakas M. C. Fujii M. Li M. Lutfi B. Kyhos J. McElroy B. (2001). High-dose intravenous immune globulin for stiff-person syndrome. *N. Engl. J. Med.* 345 1870–1876. 10.1056/nejmoa01167 11756577

[B9] Duddy M. E. Baker M. R. (2009). Stiff person syndrome. *Front. Neurol. Neurosci.* 26:147–165. 10.1159/000212375 19349711

[B10] Florea V. Rieger A. C. DiFede D. L. El-Khorazaty J. Natsumeda M. Banerjee M. N. (2017). Dose comparison study of allogeneic mesenchymal stem cells in patients with ischemic cardiomyopathy (The TRIDENT Study). *Circ. Res.* 121 1279–1290. 10.1161/circresaha.117.311827 28923793 PMC8742223

[B11] Folkers K. M. Bateman-House A. (2018). Improving expanded access in the United States: the role of the institutional review board. *Ther. Innov. Regul. Sci.* 52 285–293. 10.1177/2168479018759661 29723059

[B12] Fontaine M. J. Shih H. Schafer R. Pittenger M. F. (2016). Unraveling the mesenchymal stromal cells’ paracrine immunomodulatory effects. *Transfus. Med. Rev.* 30 37–43. 10.1016/j.tmrv.2015.11.004 26689863

[B13] Galipeau J. Sensebe L. (2018). Mesenchymal stromal cells: clinical challenges and therapeutic opportunities. *Cell Stem Cell* 22 824–833. 10.1016/j.stem.2018.05.004 29859173 PMC6434696

[B14] Gersey Z. C. Burks S. S. Anderson K. D. Dididze M. Khan A. Dietrich W. D. (2017). First human experience with autologous Schwann cells to supplement sciatic nerve repair: report of 2 cases with long-term follow-up. *Neurosurg. Focus* 42:E2. 10.3171/2016.12.FOCUS16474 28245668

[B15] Gershanik O. S. (2009). Stiff-person syndrome. *Parkinsonism Relat. Disord.* 15 (Suppl. 3) S130–S134. 20082974 10.1016/S1353-8020(09)70799-0

[B16] Glassberg M. K. Minkiewicz J. Toonkel R. L. Simonet E. S. Rubio G. A. DiFede D. (2017). Allogeneic human mesenchymal stem cells in patients with idiopathic pulmonary fibrosis via intravenous delivery (AETHER): a phase I safety clinical trial. *Chest* 151 971–981. 10.1016/j.chest.2016.10.061 27890713 PMC6026255

[B17] Guenard V. Kleitman N. Morrissey T. K. Bunge R. P. Aebischer P. (1992). Syngeneic Schwann cells derived from adult nerves seeded in semipermeable guidance channels enhance peripheral nerve regeneration. *J. Neurosci.* 12 3310–3320. 10.1523/jneurosci.12-09-03310.1992 1527582 PMC6575729

[B18] Hare J. M. Bolli R. Cooke J. P. Gordon D. J. Henry T. D. Perin E. C. (2013). Phase II clinical research design in cardiology: learning the right lessons too well: observations and recommendations from the Cardiovascular Cell Therapy Research Network (CCTRN). *Circulation* 127 1630–1635. 10.1161/circulationaha.112.000779 23588961 PMC3967793

[B19] Hare J. M. DiFede D. L. Rieger A. C. Florea V. Landin A. M. El-Khorazaty J. (2017). Randomized Comparison of Allogeneic Versus Autologous Mesenchymal Stem Cells for Nonischemic Dilated Cardiomyopathy: POSEIDON-DCM Trial. *J. Am. Coll. Cardiol.* 69 526–537. 27856208 10.1016/j.jacc.2016.11.009 PMC5291766

[B20] Hare J. M. Fishman J. E. Gerstenblith G. DiFede Velazquez D. L. Zambrano J. P. Suncion V. Y. (2012). Comparison of allogeneic vs autologous bone marrow-derived mesenchymal stem cells delivered by transendocardial injection in patients with ischemic cardiomyopathy: the POSEIDON randomized trial. *JAMA* 308 2369–2379. 10.1001/jama.2012.25321 23117550 PMC4762261

[B21] Heldman A. W. DiFede D. L. Fishman J. E. Zambrano J. P. Trachtenberg B. H. Karantalis V. (2014). Transendocardial mesenchymal stem cells and mononuclear bone marrow cells for ischemic cardiomyopathy: the TAC-HFT randomized trial. *JAMA* 311 62–73. 24247587 10.1001/jama.2013.282909 PMC4111133

[B22] Hogan M. (2016). (R)evolution: toward a new paradigm of policy and patient advocacy for expanded access to experimental treatments. *BMC Med.* 14:39. 10.1186/s12916-016-0586-6 26926908 PMC4772317

[B23] Jarow J. P. Lemery S. Bugin K. Lowy N. (2017). Ten-year experience for the center for drug evaluation and research, part 2: FDA’s role in ensuring patient safety. *Ther. Innov. Regul. Sci.* 51 246–249. 10.1177/2168479016679214 28553566 PMC5443559

[B24] Jarow J. P. Lemery S. Bugin K. Khozin S. Moscicki R. (2016). Expanded access of investigational drugs: the experience of the center of drug evaluation and research over a 10-year period. *Ther. Innov. Regul. Sci.* 50 705–709. 10.1177/2168479016656030 27917324 PMC5135086

[B25] Jerusalem G. Mariani G. Ciruelos E. M. Martin M. Tjan-Heijnen V. C. Neven P. (2016). Safety of everolimus plus exemestane in patients with hormone-receptor-positive, HER2-negative locally advanced or metastatic breast cancer progressing on prior non-steroidal aromatase inhibitors: primary results of a phase IIIb, open-label, single-arm, expanded-access multicenter trial (BALLET). *Ann. Oncol.* 27 1719–1725. 10.1093/annonc/mdw249 27358383

[B26] Jin S. Pazdur R. Sridhara R. (2017). Re-evaluating eligibility criteria for oncology clinical trials: analysis of investigational new drug applications in 2015. *J. Clin. Oncol.* 35 3745–3752. 10.1200/jco.2017.73.4186 28968168 PMC5692723

[B27] Knoepfler P. S. (2015). From bench to FDA to bedside: US regulatory trends for new stem cell therapies. *Adv. Drug Deliv. Rev.* 8 192–196. 10.1016/j.addr.2014.12.001 25489841 PMC4398607

[B28] Levi A. D. Burks S. S. Anderson K. D. Dididze M. Khan A. Dietrich W. D. (2016). The use of autologous Schwann cells to supplement sciatic nerve repair with a large gap: first in human experience. *Cell Transplant.* 25 1395–1403. 10.3727/096368915x690198 26610173

[B29] Lindsay R. M. (1988). Nerve growth factors (NGF, BDNF) enhance axonal regeneration but are not required for survival of adult sensory neurons. *J. Neurosci.* 8 2394–2405. 10.1523/jneurosci.08-07-02394.1988 3249232 PMC6569525

[B30] Mendicino M. Bailey A. M. Wonnacott K. Puri R. K. Bauer S. R. (2014). MSC-based product characterization for clinical trials: an FDA perspective. *Cell Stem Cell* 14 141–145. 10.1016/j.stem.2014.01.013 24506881

[B31] Otto D. Unsicker K. Grothe C. (1987). Pharmacological effects of nerve growth factor and fibroblast growth factor applied to the transectioned sciatic nerve on neuron death in adult rat dorsal root ganglia. *Neurosci. Lett.* 83 156–160. 10.1016/0304-3940(87)90233-3 3441295

[B32] Pagano M. B. Murinson B. B. Tobian A. A. King K. E. (2014). Efficacy of therapeutic plasma exchange for treatment of stiff-person syndrome. *Transfusion* 54 1851–1856. 10.1111/trf.12573 24527774

[B33] Pittenger M. F. D. D. Peault B. M. Phinney D. G. Hare J. M. Caplan A. I. (2019). Mesenchymal stem cell perspective- cell biology to clinical progress. *NPJ Regen. Med.* 4:22. 31815001 10.1038/s41536-019-0083-6 PMC6889290

[B34] Pittenger M. F. Le Blanc K. Phinney D. G. Chan J. K. (2015). MSCs: scientific support for multiple therapies. *Stem Cells Int.* 2015:280572. 26294916 10.1155/2015/280572 PMC4532948

[B35] Premer C. Blum A. Bellio M. A. Schulman I. H. Hurwitz B. E. Parker M. (2015). Allogeneic mesenchymal stem cells restore endothelial function in heart failure by stimulating endothelial progenitor cells. *EBioMedicine* 2 467–475. 10.1016/j.ebiom.2015.03.020 26137590 PMC4485912

[B36] Puthumana J. Miller J. E. Kim J. Ross J. S. (2018). Availability of investigational medicines through the US food and drug administration’s expanded access and compassionate use programs. *JAMA Netw. Open* 1:e180283. 10.1001/jamanetworkopen.2018.0283 30646072 PMC6324420

[B37] Rich K. M. Alexander T. D. Pryor J. C. Hollowell J. P. (1989). Nerve growth factor enhances regeneration through silicone chambers. *Exp. Neurol.* 105 162–170. 10.1016/0014-4886(89)90115-5 2753116

[B38] Rich K. M. Luszczynski J. R. Osborne P. A. Johnson E. M. Jr. (1987). Nerve growth factor protects adult sensory neurons from cell death and atrophy caused by nerve injury. *J. Neurocytol.* 16 261–268. 10.1007/bf01795309 3625240

[B39] Rieger A. C. Myerburg R. J. Florea V. Tompkins B. A. Natsumeda M. Premer C. (2019). Genetic determinants of responsiveness to mesenchymal stem cell injections in non-ischemic dilated cardiomyopathy. *EBioMedicine* 48 377–385. 10.1016/j.ebiom.2019.09.043 31648988 PMC6838383

[B40] Santamaria A. J. Benavides F. D. DiFede D. L. Khan A. Pujol M. V. Dietrich W. D. (2018). Clinical and neurophysiological changes after targeted intrathecal injections of bone marrow stem cells in a c3 tetraplegic subject. *J. Neurotrauma* 36 500–516. 10.1089/neu.2018.5716 29790404

[B41] Schulman I. H. Hare J. M. (2018). “Mesenchymal Stromal Cells as a Therapeutic Intervention,” in *Stromal Cells*, ed. Valarmathi M. (London: IntechOpen).

[B42] Schulman I. H. Balkan W. Saltzman R. DaFonseca D. Caceres L. Delgado C. (2018a). “Unique Aspects of the Design of Phase I/II Clinical Trials of Stem Cell Therapy,” in *The Management of Clinical Trials*, ed. Abdeldayem H. (London: IntechOpen).

[B43] Schulman I. H. Khan A. Hare J. M. (2018b). Interdisciplinary Stem Cell Institute at the University of Miami Miller School of Medicine. *Circ. Res.* 123 1030–1032. 30355165 10.1161/CIRCRESAHA.118.310426 PMC6289183

